# Three-dimensional dose prediction based on deep convolutional neural networks for brain cancer in CyberKnife: accurate beam modelling of homogeneous tissue

**DOI:** 10.1093/bjro/tzae023

**Published:** 2024-08-16

**Authors:** Yuchao Miao, Ruigang Ge, Chuanbin Xie, Xiangkun Dai, Yaoying Liu, Baolin Qu, Xiaobo Li, Gaolong Zhang, Shouping Xu

**Affiliations:** National Cancer Center/National Clinical Research Center for Cancer/Cancer Hospital, Chinese Academy of Medical Sciences and Peking Union Medical College, Beijing, 100021, China; Department of Radiation Oncology, Fujian Medical University Union Hospital, Fuzhou, Fujian, 350001, China; Department of Radiation Oncology, the First Medical Center of the People’s Liberation Army General Hospital, Beijing, 100853, China; Department of Radiation Oncology, the First Medical Center of the People’s Liberation Army General Hospital, Beijing, 100853, China; Department of Radiation Oncology, the First Medical Center of the People’s Liberation Army General Hospital, Beijing, 100853, China; School of Physics, Beihang University, Beijing, 102206, China; Department of Radiation Oncology, the First Medical Center of the People’s Liberation Army General Hospital, Beijing, 100853, China; Department of Radiation Oncology, Fujian Medical University Union Hospital, Fuzhou, Fujian, 350001, China; School of Physics, Beihang University, Beijing, 102206, China; National Cancer Center/National Clinical Research Center for Cancer/Cancer Hospital, Chinese Academy of Medical Sciences and Peking Union Medical College, Beijing, 100021, China

**Keywords:** dose prediction, CyberKnife, deep CNNs, brain cancer

## Abstract

**Objectives:**

Accurate beam modelling is essential for dose calculation in stereotactic radiation therapy (SRT), such as CyberKnife treatment. However, the present deep learning methods only involve patient anatomical images and delineated masks for training. These studies generally focus on traditional intensity-modulated radiation therapy (RT) plans. Nevertheless, this paper aims to develop a deep CNN-based method for CyberKnife plan dose prediction about brain cancer patients. It utilized modelled beam information, target delineation, and patient anatomical information.

**Methods:**

This study proposes a method that adds beam information to predict the dose distribution of CyberKnife in brain cases. A retrospective dataset of 88 brain and abdominal cancer patients treated with the Ray-tracing algorithm was performed. The datasets include patients’ anatomical information (planning CT), binary masks for organs at risk (OARs) and targets, and clinical plans (containing beam information). The datasets were randomly split into 68, 6, and 14 brain cases for training, validation, and testing, respectively.

**Results:**

Our proposed method performs well in SRT dose prediction. First, for the gamma passing rates in brain cancer cases, with the 2 mm/2% criteria, we got 96.7% ± 2.9% for the body, 98.3% ± 3.0% for the planning target volume, and 100.0% ± 0.0% for the OARs with small volumes referring to the clinical plan dose. Secondly, the model predictions matched the clinical plan’s dose-volume histograms reasonably well for those cases. The differences in key metrics at the target area were generally below 1.0 Gy (approximately a 3% difference relative to the prescription dose).

**Conclusions:**

The preliminary results for selected 14 brain cancer cases suggest that accurate 3-dimensional dose prediction for brain cancer in CyberKnife can be accomplished based on accurate beam modelling for homogeneous tumour tissue. More patients and other cancer sites are needed in a further study to validate the proposed method fully.

**Advances in knowledge:**

With accurate beam modelling, the deep learning model can quickly generate the dose distribution for CyberKnife cases. This method accelerates the RT planning process, significantly improves its operational efficiency, and optimizes it.

## Introduction

Radiation therapy (RT) is one of the most common cancer treatment methods. However, there are dose-sensitive organs at risk (OARs) around the gross tumour volume (GTV) that need to be protected during the treatment process, which causes high complexity for treatment planning.^1^ Generally, planners iteratively update plan parameters through a treatment planning system (TPS) to optimize the dose delivery, such as the trade-offs between the dose target coverage and the OARs’ dose limitations. The optimization process is time-consuming (from hours to days) and highly relies on the planner’s experience.^2^^,^^3^

Although advances in TPS have already reduced human intervention with the accelerated process, the degree of automation for planning still needs improvement. Moreover, the plan quality is subject to institutional guidance, planner skills, and personal clinical experiences. TPS typically utilizes dose-volume histograms (DVHs) as optimization guidance, which cannot reflect the spatial variability of 3-dimensional (3D) dose distribution within the patient’s body (regions of interest, ROIs) and are “blind” to nondelineated OARs.^4^ Consequently, the conventional RT planning process may remain lengthy and tedious, hindering the input of adaptive strategies and delaying treatment delivery, thus compromising patient care.^5^

Numerous deep learning (DL)-based algorithms have recently been developed, and data-driven knowledge-based planning (KBP) techniques have become famous.^6^ Compared to manual human adjustments, KBP can reduce the planning time from several hours to less than an hour. Although the model generated only partially meets the physician’s requirements, utilizing the method to initialize the planning process before manual adjustments may save time.^7^ Deep neural networks hold robust data (matrix) fitting capability. Thus, they have been used to perform RT treatment planning tasks in recent years.^6^^,^^8^ KBP’s primary DL models include U-Net and Generative Adversarial Network.

However, the mainstream KBP methods need to use the patient anatomy (e.g., CT) and binary masks. Furthermore, some studies added the beam information. Barragán-Montero et al^9^ proposed 2 models, the anatomy-only model and the anatomy model with added beams, for studying the impact of variable beam configurations on dose prediction in lung cancer patients undergoing intensity-modulated radiation therapy (IMRT). Zhou et al^10^ analysed postoperative cases of IMRT rectal cancer and developed a DL model called 3D U-Res-Net_B. This model utilized 3D matrices of CT images, contours, and beam configurations to predict 3D dose distributions. Peng et al^11^ examined the influence of beam angle, beam number, and patient setup on a DL model for predicting dose distributions in cervical cancer patients undergoing IMRT.

Previously, the DL method has been used in several studies for different site dose prediction of IMRT or volumetric modulated arc therapy, such as cervical,^12^ prostate,^13^^,^^14^ head and neck,^15^^,^^16^ and lung regions.^9^ It is worth mentioning that sound, and clinically consistent DL-based dose prediction results have been obtained. Artificial intelligence application in clinical practice is still limited to conventional linear accelerators; thus, more research on high-precision stereotactic radiation therapy (SRT) devices is needed. Hitherto, most dose prediction studies only focus on single-site treatment, providing much repetitive work.^17–19^ Based on the device’s uniqueness, the CyberKnife system can accurately deliver high radiation doses to the target volume while protecting surrounding tissues reasonably well. The characteristics of less fractionation and high-dose delivery can sufficiently reduce the course of treatment time.^20–23^

This work presents a DL method for beam modelling accomplished brain cancer dose prediction. Our approach transforms the beam information from CyberKnife TPS into 3D beam data through a series of physical and mathematical principles. The beam matrix reflects the relationship between the beam setups and dose delivery, which provides essential information for 3D dose prediction. We also introduced an additional halo contour (Rings) around the target area for typical consideration of the effect from the original CT scans and did 4 sets of comparative tests. Finally, a series of evaluations, including comparing gamma passing rates, DVHs, and 3D dose differences, proved our study’s feasibility. It is the first CyberKnife-based dose prediction study, which provides a method for planners to incorporate the necessary trade-offs in their decision-making and to find a balance between desired and achievable goals.

## Materials and methods

### Materials

In this study, we retrospectively collected a dataset of 112 patients with brain (mainly intracranial lesions) and abdominal (mainly liver cancers) tumours who had received CyberKnife treatment from 2018 to 2021 at our hospital. The CT images of all patients in this study were acquired using the same SIEMENS Somatom Definition AS64 CT scanner from Germany. All patient RT doses were calculated using the TPS build-in Ray-tracing algorithm (Accuray, System Version: CyberKnife 3.3.0, Software Version: Multiplan 4.0.2, USA). According to the AAPM TG101,^24^ the prescription doses to treat brain cancer cases are 21-60 Gy, and for abdominal tumours, the doses range from 42.5 to 57.5 Gy. Sometimes, insufficient target coverage is clinically acceptable to ensure limited doses of critical OARs; moreover, some brain cases had multiple targets that had been treated, and here, we only consider RT plans for single lesions. After careful selection, 88 patients were finally selected, including 54 brain (intracranial: 50; parotid: 4) and 34 abdominal cases (liver: 29; pancreas: 5) and more patient characteristics are shown in Table S1.

These patients were randomly divided into 68, 6, and 14 brain cases for the model’s training, validation, and testing, respectively. In this study, high-quality abdominal cases were fed into the network training to improve the generalization of the model so that the model is more likely to “learn” the mapping between the location and values of the dose matrix and the beam matrix established by the beam model in 3 dimensions of space. In addition, it should be noted that the patient dataset involved in medical ethical review is listed separately at the end of the article, and they were used only for academic scientific research.

### Methods

#### Overview

**Figure 1.**
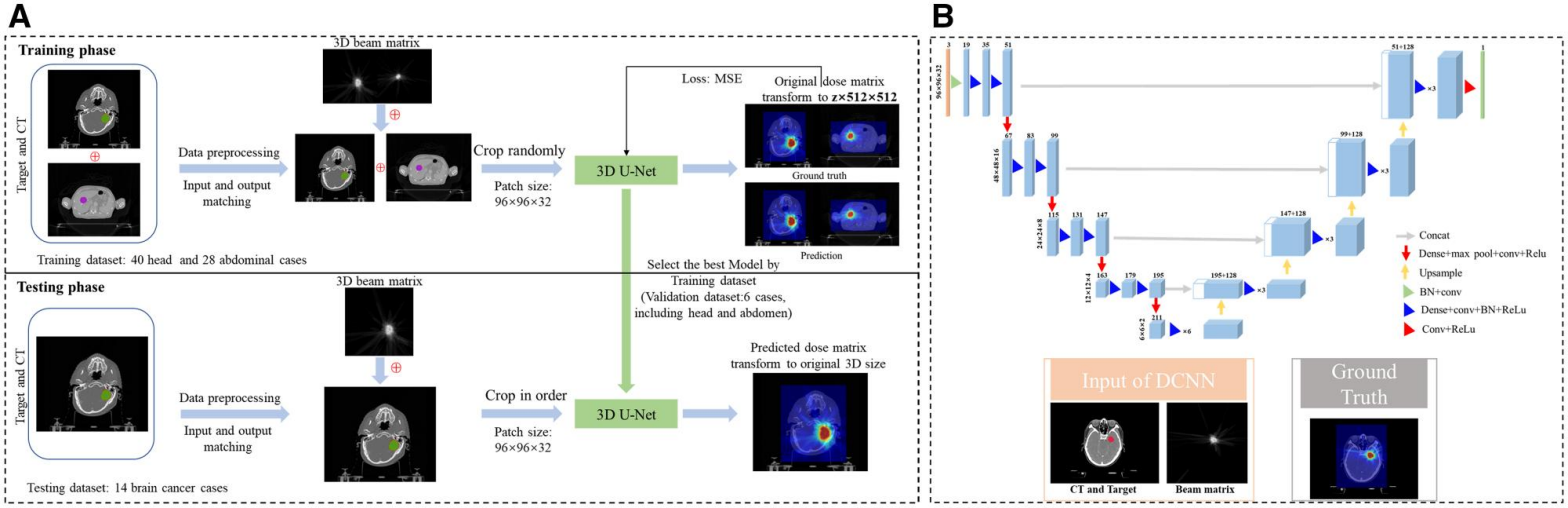
The overall workflow of our dose prediction method in the beam modelling (A) and 3D U-Net architecture (B).

Figure 1 shows the general workflow of our method. First, we extracted the target volume (PTV/GTV) for all cases with binary masks. Then, we paired the extracted target masks and the corresponding CT images as the network input and the 3D dose matrix as the network output. Also, the dose matrix slice data (axial plane) were uniformly stitched and cropped to 512 × 512. Finally, we added the beam data after beam encoding along the training channel (the specific algorithm procedure of beam encoding was described in detail below). We fed the compacted data to the 3D U-Net.^25^ Its well-established structure is familiar, so we will only briefly introduce and focus on the essential details of the 3D U-Net model constructed. Illustrated in Figure 1, the model consists of 3 input channels, with a 96 × 96 × 32 randomly sampled 3D matrix. It employs 4-fold upsampling and downsampling and incorporates a densely connected layer at each hierarchical level, creating a “dense structure.” Each dense block encompasses a ReLU activation process, followed by convolution (3 × 3 × 3 kernel size), batch normalization, and connection to the previous layer. Zero padding is applied to each convolution, with 16 channels allocated to each layer. The 3D U-Net model undergoes downsampling via max pooling (2 × 2 × 2 kernel size) and symmetrical upsampling via deconvolution (2 × 2 × 2 kernel size, channel = 128). The final hierarchical layer of the convolution generates a single-channel matrix, which serves as the output matrix.

Given that the validation set was employed for model selection, but due to the small amount of data used for validation, we selected the model that passed the validation of the dataset and performed the best on the training set. Furthermore, 14 eligible brain cancer cases were selected from the randomized grouping phase of the data as the test set, which was used exclusively for testing purposes. We selected the optimal model in the testing phase, inferred the corresponding 3D dose maps, and saved the data into (.dcm) files. Afterward, we performed qualitative and quantitative analysis on the predicted dose.

#### Beam modelling

We draw a simplified schematic of the specific beam modelling process based on the CyberKnife RT principle and setups.

*Step 1*: The CT images were used to create a 3D phantom, capturing all voxels coordinate information (*x*, *y*, *z*). The target volume was divided into multiple “small sub-targets,” with the specific count determined by the number of delivery beams employed.

*Step 2*: We streamlined the modelling process for the linear accelerator (Linac) treatment head by treating the initial X-ray excitation point as a dummy “radioactive source.” Following the manufacturer’s specifications, we set the distance from this source to the beam exit at the lower end of the secondary collimator (treatment head) to approximately 40 cm. This physical placement was subsequently mapped to its corresponding position within the modelled beam matrix.

*Step 3*: We extracted 4 essential 3D coordinate parameters for the treatment head and target point from the TPS RTplan file. These parameters included the size of the secondary collimator and the number of the beam’s MUs (Monitor units). To standardize, we normalized the secondary collimator’s projection at the target volume to 1 within the projection area and 0 outside it. Leveraging this normalized collimator projection, we multiplied it by the MUs.

*Step 4*: We approximated the beam tensor angle for treatment delivery using the formula arctan((cone/4)/L_b-source_), where L_b-source_ represents the distance from the source to the treatment head. This data was also stored within the 3D matrix for reference.

*Step 5*: Coordinates *P* (*x*, *y*, *z*) were set to denote any point in the CT phantom, and the tensor angle of the vector composed of the treatment head to point *P* concerning the beam centre axis. A coordinate *P* within the treatment head tensor angle would be considered a beam passing through and assigned a value. The assignment principle uses a specific beam’s MUs divided by the number of non-zero MUs doing the smallest MUs during treatment, multiplied by 1. Judgment: ∠(vector(b-point) and vector(beam)) <= beam tensor angle. We modelled each point in the beam matrix using the formula:

beam matrix=∑j=1n∑i=1m(Oi+T0,1×MUjmin{MU1,MU2…MUn}∏voxeli)where, m=CT voxel number, and ∠(bjpi→,bjaj→)≤arctan(cone/4Lb-source)∏voxeli=the CT voxel iO1=zero matrix, Oi+1=Oi+T0,1×MUjmin{MU1,MU2…MUn}∏voxeliMUj=beam or MUj, n=beam numberT=target area

and saved the result as the “beam model matrix.”

*Step 6*: Parallel multithreaded CPUs were utilized to speed up the beam encoding process. It takes about 2 h or less for a single case.

**Figure 2.**
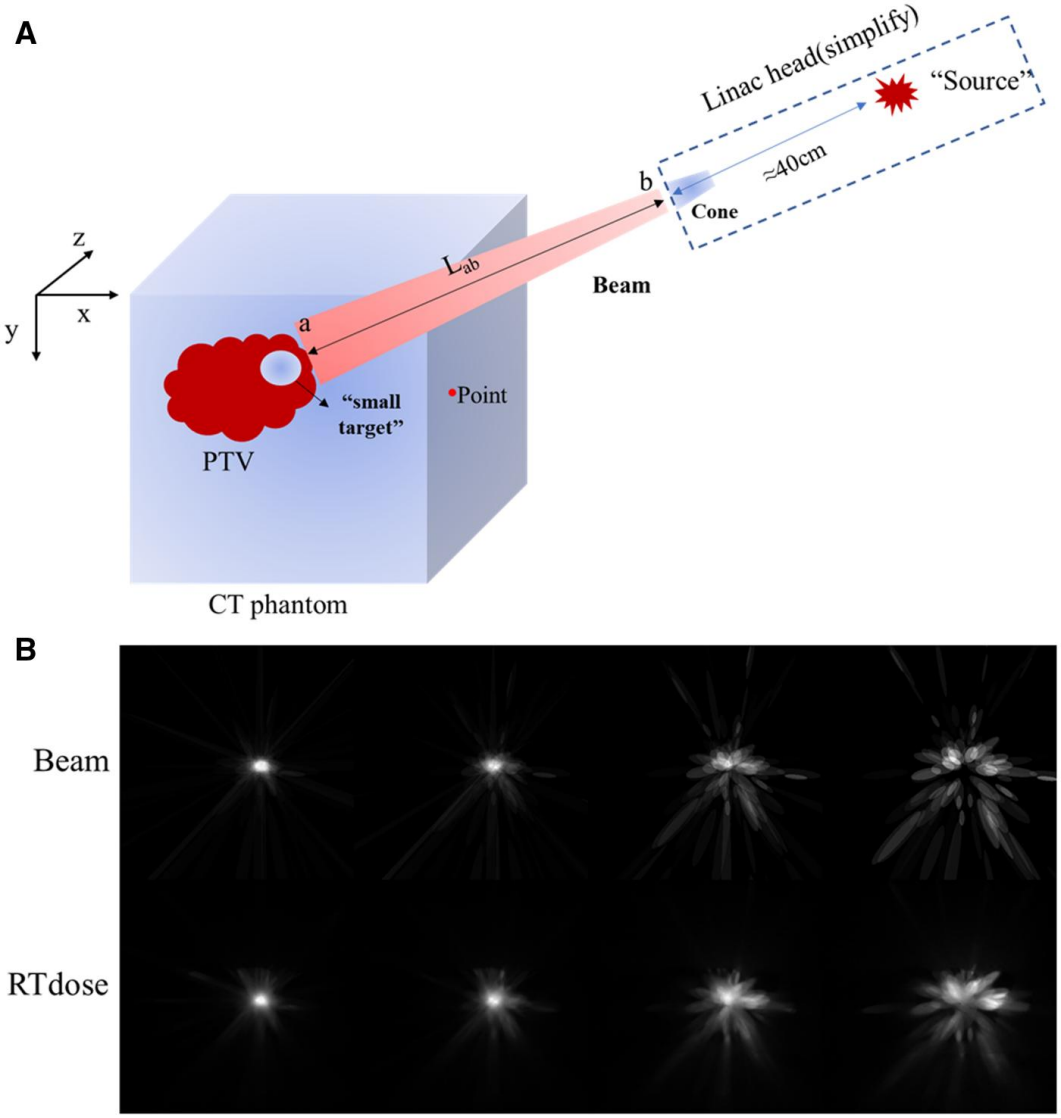
Simplified diagram of beam modelling and comparison of dose results. (A) Simplified schematic diagram of the treatment process of CyberKnife. (B) Transversal visualization of beam encoding and dose matrices.

Python code entirely accomplished the file reading, conversion, beam coding and matrix transformation. Figure 2B compares the beam coding and dose distribution matrices. They are visually similar, but beam coding benefits 3D dose prediction.

#### Training and validating the model

The training loss between the predicted dose, *D*_pre_, and the ground truth dose, *D*_gt_, was the mean square error given in the following equation.^15^

$$\mathrm{loss}(\mathrm{pre},\mathrm{clinical})=\frac{1}{n}\sum_{i=1}^{n}{\left(D_{\mathrm{pre}}^{i}-D_{\mathrm{gt}}^{i}\right)}^{2}$$

where *i* denotes the voxel index, *n* denotes the number of voxels. The 3D U-Net training was performed on a server with a 72-core Intel(R) Xeon(R) Gold 6139M CPU @ 2.30 GHz and 4 of 24 GB NVIDIA GeForce RTX 3090 s, with TensorFlow-GPU version 2.4.0 platform. The training utilized the Adam stochastic optimization algorithm with an initial learning rate of 1e^−4^. (The learning rate decayed from 1e^−4^ to 1e^−6^ during CNN network training.) One hundred data slices were randomly collected for each case, and the batch size was set to 2. Due to the addition of dense blocks to the network,^26^^,^^27^ the training process was extended. The model training was stopped after 60 epochs, while the loss value on training and validation data tends to be stable. These 60 epochs of training took about 10 days. However, due to the limited size of the validation set, its loss values display a slight fluctuation around the training set. In this context, we select the model with the lowest loss function value in the training set as the designated “best model.”

#### Model evaluation

Four different methods, TB (Target and beam), TBR (TB and rings), CT+TB, and CT+TBR, were evaluated in the 14 brain cancer test cases. The letters represent different data fed into the network for training, such as T: Target delineation, B: Beam matrix, and R: rings (halo ring). The CT refers to the Computed Tomography images of the patient. We used 4 composite metrics to evaluate the predicted dose distribution. First, we calculated the 3D gamma passing rates for the target and OARs between the predicted and ground truth doses. Second, the mean absolute errors of the maximum and mean doses of the OARs between the predicted and ground truth values were calculated.

Furthermore, we also compared the mean dose, D_98_, D_95_, D_90_, and D_50_ differences for the target volume. Third, we calculated the absolute dose differences for each voxel between the predicted and ground truth doses. Finally, we compared the DVHs for each ROI in brain cancer patients. The standard deviation was considered for the first 3 metrics.

The new conformity index (nCI), homogeneity index (HI), and gradient index (GI) were calculated using the formula below^28^^,^^29^:

nCI=(TV×VRI)/(TVRI×TVRI)HI=D5/D95GI=volume of 50% isodose line/volume of prescription isodose line

In the first formula above, *TV* represents the target volume (PTV), *TV*_RI_ is the volume of PTV covered by the prescription dose, and *V*_RI_ is the entire volume covered by the prescription dose. In the formula below, *D*_5_ and *D*_95_ denote the doses covering 5% and 95% of the PTV volume.

## Results

Four methods (input TB, TBR, and the proposed CT+TB/CT+TBR) were evaluated on the 14 CyberKnife brain cancer cases tested. First, we calculated the 3D gamma passing rates of the predicted and clinical doses in the target volumes (PTV and GTV) (as shown in Table 1, calculation tool: open-source software 3D slicer [version 4.11],^30^ the calculation criteria: 3 mm/3%, 3 mm/2% and 2 mm/2%).^31^ Horizontally, the comparison shown in Table 1 indicates that our method (CT+TB and CT+TBR groups) produced accurate overall dose prediction results in the body and target area with excellent robustness, both in terms of mean and standard deviation, for both the body (98%, 3 mm/3%) and the target area (99%, 3 mm/3%). Longitudinally, the prediction results for the former 2 experimental bodies (TB and TBR groups) were more robust than those for the target area. Meanwhile, the 3 parameters of the nCI, HI and GI in TBR, CT+TB, and CT+TBR groups within the target area agreed well with the clinical plan. However, the latter 2 were better (the prediction results in the TB group were not good in the target area, and it was impossible to count the above 3 target area indicators).

**Table 1.** The gamma passing rates and critical indicators of the target area in brain cancer cases (mean ± SD).

	Criteria	TB	TBR	CT+TB	CT+TBR
**Body (%)**	3 mm/3%	87.1 ± 5.9	98.6 ± 1.6	98.6 ± 1.6	99.5 ± 0.5
	3 mm/2%	86.6 ± 6.1	98.4 ± 1.7	98.4 ± 1.7	99.4 ± 0.6
	2 mm/2%	78.3 ± 9.2	96.0 ± 3.4	96.7 ± 2.9	98.3 ± 1.3
**PTV (%)**	3 mm/3%	53.7 ± 12.4	94.6 ± 7.1	99.8 ± 0.3	99.6 ± 0.7
	3 mm/2%	52.9 ± 12.4	94.1 ± 7.4	99.7 ± 0.5	99.3 ± 1.1
	2 mm/2%	37.0 ± 9.5	85.0 ± 13.4	98.3 ± 3.0	98.1 ± 2.1
**GTV (%)**	3 mm/3%	39.1 ± 13.3	93.2 ± 8.5	99.8 ± 0.4	99.4 ± 1.0
	3 mm/2%	38.3 ± 13.3	92.5 ± 8.9	99.6 ± 0.7	99.0 ± 1.5
	2 mm/2%	20.1 ± 7.8	80.9 ± 16.0	97.8 ± 3.7	97.3 ± 3.2
**(nCI, HI, and GI)**					

	**Clinical planning**		**TBR**	**CT+TB**	**CT+TBR**

**nCI**	1.02 ± 0.01		1.01 ± 0.01	1.02 ± 0.01	1.02 ± 0.01
**HI**	1.31 ± 0.04		1.36 ± 0.05	1.34 ± 0.05	1.31 ± 0.05
**GI**	1.02 ± 0.01		1.01 ± 0.01	1.02 ± 0.01	1.02 ± 0.01

For an mm/b% gamma passing rate, voxel “pass” if at least one voxel in the predicted dose distribution is within an mm range of that voxel and the dose received is within the ±b% range of that voxel dose value in the clinical plan. The passing rate represents the percentage of the passing voxels. The larger the value is, the better the predicted result is. The target area’s nCI, HI, and GI are critical indicators to evaluate plan quality in clinical practice. The closer the nCI is to 1, the better the result is.

Abbreviations: GI = gradient index; GTV = gross tumour volume; HI = homogeneity index; nCI = new conformity index; PTV = planning target volume.

In summary, the 4 groups’ results suggest that the prediction results of our proposed method performed better predictions in body and target areas. Moreover, if the target halos were added right at the target delineation (e.g., CT+TB vs CT+TBR), this would improve the dose prediction for the body and OARs (as shown in Table 2). In addition, comparing the TB and CT+TB groups, it is easy to see that CT images as anatomical information are essential inputs to the network. Meanwhile, comparing the TB and TBR groups, we also found that with the addition of target halo rings, the prediction results of the TBR group were significantly improved, especially in the target area.

**Table 2.** The gamma passing rates of OARs in brain cancer cases (mean ± SD).

	Criteria	TB	TBR	CT+TB	CT+TBR
**Eye_L (%)**	2 mm/2%	100.0 ± 0.0	100.0 ± 0.0	100.0 ± 0.0	100.0 ± 0.0
**Eye_R (%)**	2 mm/2%	100.0 ± 0.0	100.0 ± 0.0	100.0 ± 0.0	100.0 ± 0.0
**Lens_L (%)**	2 mm/2%	100.0 ± 0.0	100.0 ± 0.0	100.0 ± 0.0	100.0 ± 0.0
**Lens_R (%)**	2 mm/2%	100.0 ± 0.0	100.0 ± 0.0	100.0 ± 0.0	100.0 ± 0.0
**Optic Nerve_L (%)**	2 mm/2%	98.6 ± 3.1	99.4 ± 2.4	98.8 ± 2.8	99.6 ± 1.4
**Optic Nerve_R (%)**	2 mm/2%	99.1 ± 2.2	99.9 ± 0.3	99.4 ± 1.4	100.0 ± 0.2
**Optic Chiasm (%)**	2 mm/2%	97.5 ± 5.5	99.9 ± 0.4	98.9 ± 2.4	99.0 ± 3.2
**Brainstem (%)**	2 mm/2%	93.6 ± 7.7	99.4 ± 0.9	97.7 ± 2.0	98.7 ± 2.7

Considering the small size of the OARs of the brain and the low dose, the dose threshold for calculating the gamma passing rate of the OARs is 0.5% of the reference maximum dose for the brain. Eye_L means left eye, Eye_R means right eye, and other representations in the table have similar meanings.

Abbreviation: OARs = organs at risk.

We also evaluated the differences between each OAR’s predicted and clinical dose distribution. Table 2 shows the gamma passing rates with the 2 mm/2% criteria for OARs. According to the transversal comparison in Table 2, it can be found that the 4 experimental groups did not show much difference. The OARs were predicted well, especially in the 3 groups of TBR, CT+TB, and CT+TBR. Meanwhile, the longitudinal comparison showed that the dose prediction for the miniature volume OARs (the first 4) was better than that of the immense volume OAR (brainstem), which is evident in the TB group. With the addition of CT and halo outlines, the difference in prediction results for each OAR became less pronounced.

**Figure 3.**
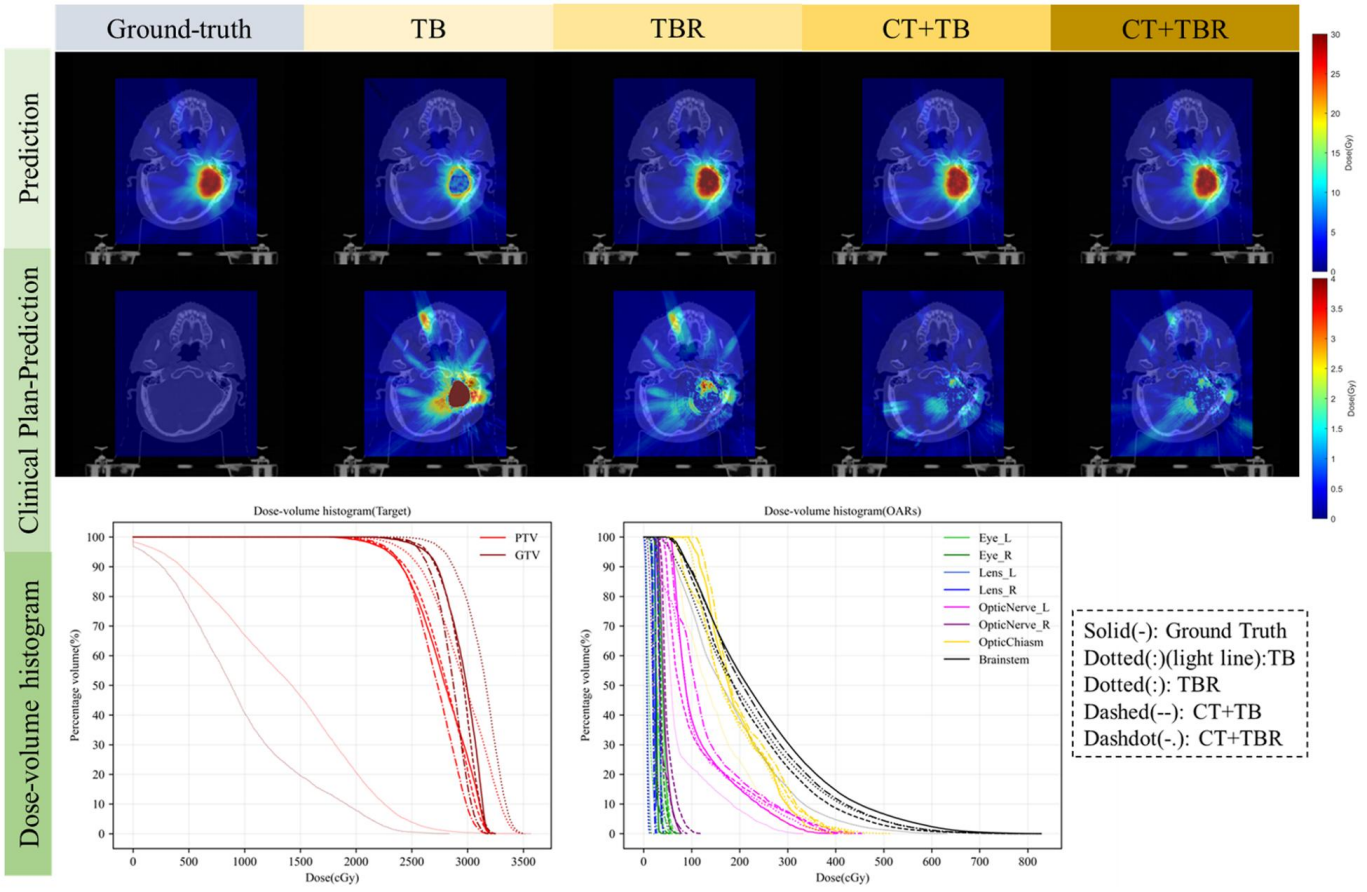
Quantitative comparison of 4 groups of experiments. In the first row, the images are the clinical plan of the brain cancer case, used as Ground truth, and the other images are the predicted results of the model. The second row shows the absolute differences between the clinical and predicted plans in each group of experiments. The last row shows the DVHs of the clinical and predicted plans in each group of experiments, containing the results of PTVs and OARs. Abbreviations: DVH = dose-volume histogram; OAR = organs at risk; PTV = planning target volume.

Based on the quantitative comparison of the 4 sets of experiments, as shown in Figure 3, we could see that regardless of the different training methods (first row), the predicted dose distribution around the target area (beam trajectory dose) was well-matched with the clinical dose. It indicated that our proposed beam-encoding-based model exhibited good performance. Moreover, we noted that the patient’s anatomical information, i.e. CT data, significantly affects the model’s prediction accuracy. Without CT information input, the dose prediction accuracy for the TB group’s target area was not so good, as seen in Table 1.

The difference between the predicted and clinical doses in the second row shows that the CT+TB and CT+TBR groups demonstrated more reasonable predictions, with the maximum dose error below 4 Gy. Most voxels had relatively small dose differences (cGy order of magnitude). Also, we noticed that the dose differences in the central area fell around the PTV. The PTV region dose prediction differences were minimal and detailed in Table 1. In addition, comparing the dose distributions of OARs in the CT+TB and CT+TBR groups, the latter predicted slightly better results than the former.

DVHs assist the clinical planning process as a significant indicator of RT planning quality assessment. The ROI dose-volume differences among the 4 experimental groups were visualized in the third row of Figure 3. The 5 different line types represent the clinical plan and the 4 comparison experiments’ dose-covered volumes (the correspondence between the curves and the experiments is noted on the right). The discrepancies in the predicted OAR doses among the 4 experiments were relatively minor and within the acceptable clinical range. In contrast, for PTV, the differences among the predictions of the 4 experiments are significant (*T*-tests were performed on the predictions of the 4 groups 2 by 2, and the *P*-values were <.05 for all but the last 2 groups).

**Figure 4.**
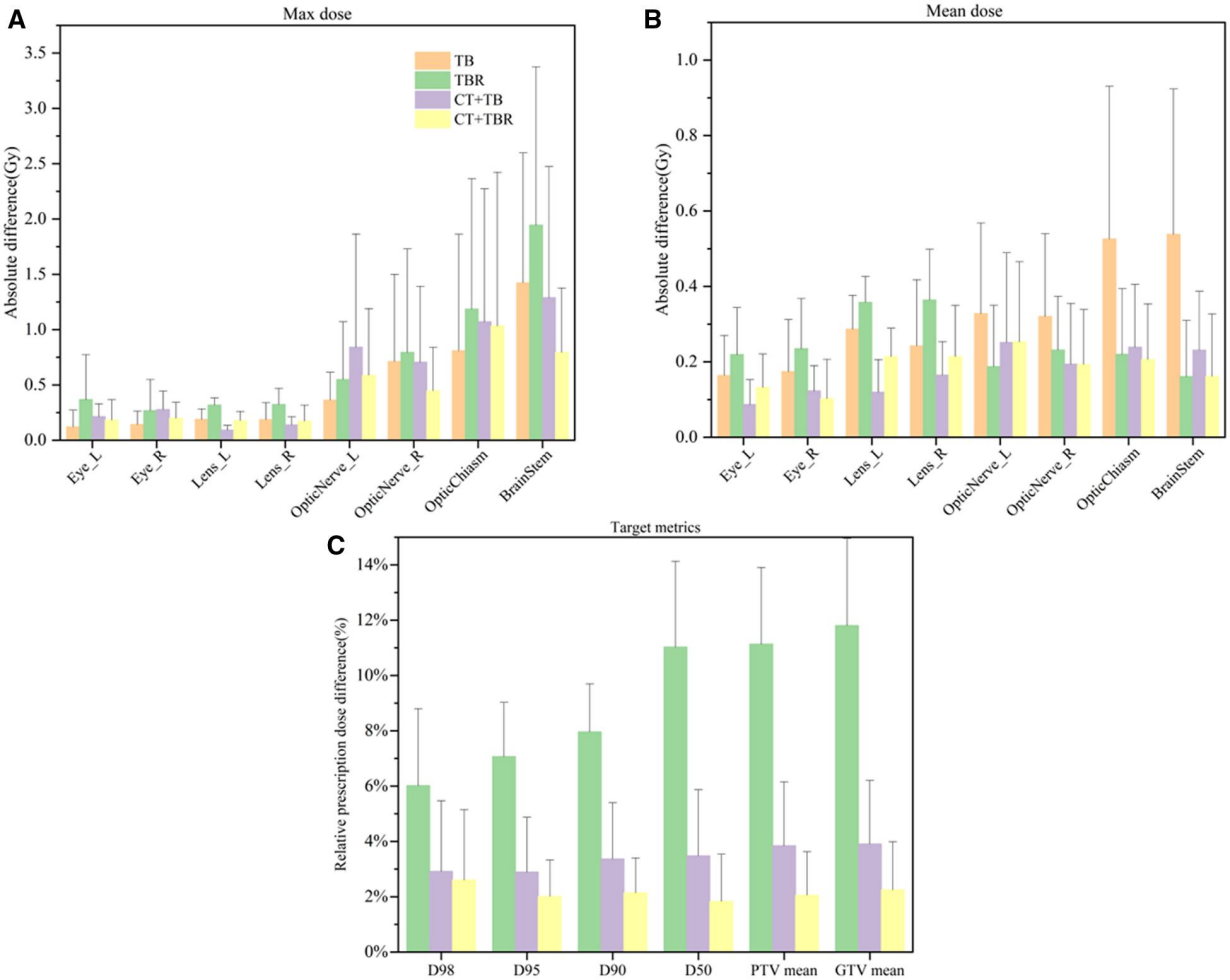
The absolute differences of dose metrics about OARs and PTV between the prediction and ground truth. (A) Maximum absolute dose differences of OARs, (B) Average absolute dose differences of OARs, and (C) Relative prescription dose differences of PTVs in brain cancer cases. Abbreviations: OAR = organs at risk; PTV = planning target volume.

To better quantify the dose differences in PTV and OARs of the 4 experimental groups, we plotted the maximum and mean dose differences separately for each OAR of brain cancer cases. The histogram also included the following metrics: D_98_, D_95_, D_90_, D_50_, and PTV_mean_/GTV_mean_ (mean PTV/GTV dose, mean dose ± SD), shown in Figure 4. Due to the suboptimal prediction of PTV in the TB group compared to the clinical plan, it was deemed inappropriate to plot it on the same axis as the other PTV metrics.

Among the 3 graphs of Figure 4, the differences between prediction and clinical results on PTVs and brainstem regions are pronounced. The maximum dose difference for OARs was within 3.0 Gy (about 1%-6% of the prescribed dose), and the mean dose difference was within 1.0 Gy (around 1%-2% of the prescribed dose) (both including SD). Further, it is easy to see that for the maximum dose difference, the smaller the volume of the OARs, the lower the dose error; for the average dose difference, there is no such feature. The differences in key metrics in the target area were generally below 1 Gy (approximately a 3% difference relative to the prescription dose).

Overall, the test set results show that our method predicted accurate patient dose distributions with excellent robustness in target areas and OARs, demonstrating its good generalizability.

## Discussion

In recent years, KBP techniques based on a priori knowledge have been well developed. In RT, neural network-based studies have become more prevalent.^32^^,^^33^ However, most DL-based dose prediction studies were previously limited to IMRT plans and were only done for a single tumour site. There are few studies on dose prediction in SRT, like CyberKnife, while most patients’ dose prediction is only for a single tumour site. Nevertheless, achieving the desired results with only anatomical information input takes work. Afterward, some papers mentioned that adding the beam information is helpful.^10^^,^^11^ In this study, we added the beam information of the RT plan for the training, finally accurately predicting the dose distribution for CyberKnife brain cancer cases. Based on accurate beam modelling, DL can quickly calculate the dose for CyberKnife patients, avoiding spending a lot of redundant TPS calculation time and significantly improving the operational efficiency of planning and optimizing the RT process.

Figure 2B illustrates that the beam matrix is consistent with the dose matrix over the whole body for the beam coding algorithm regarding the distribution of values at each slicer. Combined information is beneficial for 3D dose prediction. Compared with only the binary mask, inputting the beam matrix information makes the dose of the beam trajectory around the target more explicit. Thus, the learning difficulty was reduced, and the convergence rate of the network training was greatly accelerated (typically, the learning rate had decreased to 1e^−6^ within 20 epochs).

For the model structure of the 3D U-Net, when dealing with smaller-scale datasets (as opposed to large datasets), the typical approach is to train the model using n-fold cross-validation.^17^^,^^27^ However, in this study, the decision was made not to utilize this method due to time limitations. The model incorporates a dense connectivity mechanism, resulting in many model parameters and prolonged training time (over 10 h for a single iteration, especially during peak server usage). Adopting n-fold cross-validation would further increase the training time by a factor of n. Additionally, with the implementation of the beam modelling algorithm, the model’s loss function rapidly converges to a shallow value (within the orders of magnitude of 10^−4^-10^−5^) after just 20-30 iterations. Considering further from a dataset perspective, cross-validation aims to verify the validity and robustness of the model on all data. However, the model moderated all data except the test set several times during training. In addition, cross-validation is rarely used in DL research because our datasets are carefully chosen to be free from extreme deviations. Therefore, removing cross-validation does not have an impact on the experimental results. With the “best model” obtained through training, the network only requires the input of the target area, CT image, and beam encoding matrix to generate the patient’s planning dose within minutes.

The authors designed 4 experimental groups for model testing, the sources of which are briefly described below. The research in References 13 and 34 focuses on predicting the dose for IMRT tumour cases using delineated masks (contours) from CT images. For CyberKnife (CK) tumour cases, the authors hope that with less information input, the model can learn the patient dose field distribution and achieve twice the result with half the effort. To achieve this, we established a control group, which included CT images for independent comparison. The structure of this study is similar to that found in the work of others, where the CT data were compared separately for pre- and post-effects.^6^ This unique approach has not been attempted by researchers in the CK plan before, but it holds logical significance.

For test results of the target area, our proposed model that leverages beam-precise coding offers a marked enhancement in gamma passing rates and dose distribution maps within the PTVs compared to the mask-based model (refer to Table 1 and the second line of Figure 3). A common challenge encountered while addressing smaller PTVs, typical of brain tumours, is the potential for loss of the feature information due to multiple convolutions and downsampling when the network directly utilizes a binary mask. It could contribute to less satisfactory prediction results in the TB group. Reviewing the research in References 13 and 34, we found that the primary focus was on IMRT for prostate tumours. Here, the larger target volume of the tumour better retains the feature information through multiple convolutions, offering one explanation for the accurate prediction of IMRT patient dosing based solely on the delineated mask—an effect optimized in our TBR group for larger volume target rings. Our beam encoding transformation has expanded the PTV channel’s scope, starting with minor non-zero areas and extending across the entire image. This method prevents the loss of important information from smaller objects. However, even with these advancements, the data deficiency of CT anatomical information in treatments like SRT plans, such as CK, where small fields, multiple beams, and minimal fractions are the norm, underscore the persistent need for furnishing additional feature data for accurate learning and prediction by the network.

When considering OARs, it becomes apparent that each OAR is better predicted across all evaluated metrics (refer to Table 2, lines 1 and 3 of Figure 3 [DVH of OAR]). Notably, no significant differences are observed in the prediction results among the 3 groups: TBR, CT+TB, and CT+TBR. This outcome demonstrates the feasibility of our established method, based on accurate beam modelling, and affirms the validity of predicting the dose deposition along the beam trajectory. Additionally, we discovered that incorporating the target halo contours (Rings) into the network (comparing TB and TBR, CT+TB groups) yields results similar to those of using CT information. The larger volume of the halo rings retains specific features of the target area, even after multiple convolutional downsampling. Consequently, the network compensates for the lack of CT images. Conclusively, CT data provides indispensable feature information for network training, while the halo contours play a distinct role in enhancing the results. As a result, the prediction outcomes surpass or equal those of certain IMRT dose prediction studies.^6^^,^^7^

Meanwhile, for the statistics result of PTVs (Figure 4C), the CT+TBR group in brain cases was superior to the CT+TB group. However, its advantage was not predominant (in the order of cGy). Additional halo contours of the target area cause extra time spent and effort; however, if halo contours were included when delineating the target area, this could improve the target metrics. Therefore, the model input method of the CT+TB group is only an ideal choice for the current beam model. Comparing the results of the TB and CT+TB groups with those of the TBR and CT+TBR groups, we found that inputting the halo contours of the network could slightly improve the model’s performance. One possible reason is that the network model learns the electron density information, which is much more critical than the halo contours as an input.

To assess the generalizability of our method to RT plans involving homogeneous tumour tissues, we conducted additional testing on an external dataset of abdominal cases. The results of this test were aligned with the overall trend observed in the brain cases, thus confirming the favourable generalizability of our method. Given this article’s length constraints, we will refrain from delving into a detailed discussion.

In conclusion, our proposed method has many advantages. On the one hand, the learning process of the model becomes more efficient by introducing the beam encoding information. On the other hand, our method only changed the input data of the model training, and no modification was applied to the network structure; therefore, the model was highly compatible with many other networks. When the computational resources were insufficient, it was possible to use a 2D network for training, which minor modifications of the network parameters can accomplish. More importantly, this study may represent the first instance of dose prediction for CyberKnife that achieves optimal predictions compared to previous studies focused on dose prediction for conventional IMRT.^3^^,^^29^^,^^34^ This finding offers a novel perspective for researchers to explore the field of automated SRT planning.

However, there are still some “limitations” to our method. The beam matrix obtained by the coding algorithm of beam modelling each voxel unit is a deterministic value, and there is a certain degree of randomness concerning the dose deposition matrix, which shows some defects in the dose prediction. For example, in Table 2, the predicted dose of OARs in the TB group is not so good, where the model only inputs beam matrices and target area delineations. It did not demonstrate the same excellent performance as the other 3 groups in predicting some OAR results, especially in the brainstem. While comparing the dose maps in Figure 3, there are still multivoxel differences in the dose maps around PTV, which requires further optimization and enhancement of our subsequent algorithm for beam modelling. The authors propose an improvement strategy involving pairing the CT values from the patient’s CT image with the beam matrix. This convolution is followed by uniform normalization based on the patient’s prescribed dose. This approach showcases the randomness of the beam matrix values to some extent as they change with the CT values. The addition of this joint convolutional layer has the potential to optimize the prediction. In the future, we will develop a reverse planning system based on our existing commercial CyberKnife TPS to explore how our dose prediction method could be used for fast clinical planning.

## Conclusion

Deep learning-based methods are a promising way to obtain clinical RT doses quickly, efficiently, and accurately. The most commonly used method utilizes the anatomical image and its corresponding mask. On this basis, we transformed the CyberKnife case beam information into matrix coding and fed it into a neural network for training. More data input may reduce the learning difficulty and thus achieve a better outcome. We built a flexible and generalizable model and method that can be applied to multi-site homogeneous tissue(such as abdominal non-coplanar homogeneous tissue cancer).

## Supplementary Material

tzae023_Supplementary_Data
