# Supplementary material for: Three-dimensional dose prediction based on deep convolutional neural networks for brain cancer in CyberKnife: accurate beam modelling of homogeneous tissue
Source: BJR Open. 2024 Aug 16;6(1):tzae023. doi: 10.1093/bjro/tzae023 (PMC11364489; doi:10.1093/bjro/tzae023)
Supplement: tzae023_Supplementary_Data [file tzae023_supplementary_data.docx]

***The embedded table S1 in our manuscript files.***

**Table S1.** The model selected datasets of 88 patients, including 54 brain and 34 abdominal cases

| Site | Brain (54) | Abdominal (34) |
| --- | --- | --- |
| Gender (M/F) | M:26; F: 28 | M:25; F: 9 |
| Age(Y) | 10-83 (Median: 60) | 40-89 (Median: 65) |
| Prescription (Gy) | 21-60 | 42.5-57.5 |
| Fraction (*F*) | 3-5 | 4-5 |
| Tumor volume(cc) | 1-10 | 20-50 |
| Reference point (%) | 70-80 | 70-80 |
| Pathological site | Intracranial: 50; Parotid: 4 | Liver: 29; Pancreas: 5 |
